# Home literacy environment and early reading skills in Japanese Hiragana and Kanji during the transition from kindergarten to primary school

**DOI:** 10.3389/fpsyg.2023.1052216

**Published:** 2023-04-26

**Authors:** Takayuki Tanji, Tomohiro Inoue

**Affiliations:** ^1^Okayama University, Okayama, Japan; ^2^The Chinese University of Hong Kong, Hong Kong SAR, China

**Keywords:** home literacy environment, early literacy skills, Japanese Hiragana and Kanji, parent expectation, parent affect

## Abstract

We examined the reciprocal associations between home literacy environment (HLE) and children’s early reading skills in syllabic Hiragana and morphographic Kanji in a sample of Japanese parent–child dyads. Eighty-three children were followed from kindergarten to Grade 3 and tested on Hiragana reading accuracy in kindergarten, Hiragana word reading fluency in kindergarten and Grade 1, and Kanji reading accuracy in Grade 1 to Grade 3. Their parents answered a questionnaire about HLE [parent teaching (PT) in Hiragana and Kanji, shared book reading (SBR), and access to literacy resources (ALR)], parents’ needs for early literacy support by teachers, parents’ expectations for children’s reading skills, parents’ worry about children’s homework, and mother’s education level. Results showed first that ALR, but not PT and SBR, was associated with reading skills in Hiragana and Kanji. Second, whereas Hiragana reading in kindergarten was not associated with PT in Hiragana in kindergarten, it negatively predicted PT in Hiragana in Grade 1. However, Kanji reading accuracy was not associated with PT in Kanji across Grades 1 to 3. Third, parents’ worry was negatively associated with children’s reading performance across Grades 1 to 3 but positively associated with PT in Hiragana and Kanji. Finally, while parents’ expectations were positively associated with children’s reading performance across Grades 1 to 3, they were negatively associated with PT in Hiragana and Kanji in Grades 1 and 2. These results suggest that Japanese parents may be sensitive to both their children’s reading performance and social expectations for school achievement and adjust their involvement accordingly during the transition period from kindergarten to early primary grades. ALR may be associated with early reading development in both Hiragana and Kanji.

## 1. Introduction

Early reading skills develop rapidly at the beginning of primary school and thereafter by receiving formal literacy instruction. Although schools facilitate the development of children’s literacy skills, parental involvement and the home literacy environment are also responsible for reading development (Sénéchal and LeFevre, 2002; Niklas and Schneider, 2017; Inoue et al., 2020b; Silinskas et al., 2020; Georgiou et al., 2021). An increasing number of studies have demonstrated that home literacy environment (HLE) predicts children’s language and literacy development across a variety of languages and cultural contexts (Manolitsis et al., 2011; Niklas and Schneider, 2013; Sénéchal and LeFevre, 2014; Inoue et al., 2018a; Zhang et al., 2020). However, the majority of HLE studies have been conducted in North America and Europe with children learning to read in an alphabetic orthography, and studies in non-alphabetic orthographies are still relatively rare, except for those in Chinese (e.g., Chow et al., 2008; Deng et al., 2015; Zhang et al., 2020). In fact, to our knowledge, few studies in Japanese have examined the longitudinal links between HLE and children’s reading skills during the transition period from kindergarten to early primary grades. To better understand the effects of different aspects of HLE on children’s literacy skills, further studies in more diverse cultural contexts are needed (McBride et al., 2022). In addition, although several studies have provided evidence for the “evocative” effects of children’s literacy skills on parental teaching (i.e., early child’s literacy skills predict later parental involvement; Niklas and Schneider, 2013; Sénéchal and LeFevre, 2014; Deng et al., 2015; Inoue et al., 2018a; Silinskas et al., 2020, 2021; Georgiou et al., 2021), few have examined the longitudinal influences of reading skills in two different scripts on parent teaching simultaneously. Thus, in this study, we examined the longitudinal associations between HLE and early literacy skills in syllabic Hiragana and morphographic Kanji among Japanese children from kindergarten to Grade 3.

### 1.1. The associations between HLE and children’s early literacy skills

The HLE includes a variety of parent–child activities related to literacy (Burgess, 2002; Manolitsis et al., 2011). According to the Home Literacy Model (Sénéchal and LeFevre, 2002; Sénéchal, 2006), parent–child interactions during home literacy activities are broadly classified into two categories: code-related (formal) and meaning-related (informal) activities. Code-related activities are usually assessed with the frequency of parental teaching (PT) of letters/words, while meaning-related activities are often operationalized as the frequency of shared book reading (SBR). Previous studies have shown that (a) code-related activities are associated with word reading through their effect on letter knowledge, and (b) meaning-related activities are associated with oral languages skills, including vocabulary (Sénéchal, 2006; Silinskas et al., 2010b; Sénéchal and LeFevre, 2014; Inoue et al., 2018b, 2020b). Additionally, several studies have suggested that access to literacy resources (ALR), often operationalized with the number of children’s books at home and the frequency of library/bookstore visits, may play a unique role in children’s language and literacy development (e.g., Liu et al., 2018; Zhang et al., 2020) over and above the effects of code-related and meaning-related activities.

Previous studies have consistently indicated the influence of the three aspects of HLE on children’s language and literacy development across languages and cultural contexts (Silinskas et al., 2010b; Manolitsis et al., 2011; Sénéchal and LeFevre, 2014; Inoue et al., 2018a, 2020b). For example, Sénéchal and LeFevre (2014) found that SBR in kindergarten predicted growth in receptive vocabulary from kindergarten to Grade 1, whereas PT of reading in kindergarten predicted early literacy in kindergarten and Grade 1 and growth in word reading during Grade 1. In a cross-linguistic study with children from Grade 1 to Grade 2, Inoue et al. (2020b) examined the relationship between HLE and literacy skills across four alphabetic orthographies (English, Dutch, German, and Greek) and found that PT was associated with letter knowledge or phonological awareness in Dutch and Greek, while ALR was associated with different emergent literacy skills in all the languages (for similar findings, see Inoue et al., 2018b; Manolitsis et al., 2011; Silinskas et al., 2013, 2020). It should be noted, however, that while many studies found a positive association between parent teaching with children’s reading performance in primary school (Sénéchal and LeFevre, 2014), some others found negative associations between parent teaching and children’s reading (Manolitsis et al., 2013; Silinskas et al., 2021). This discrepancy in the findings of previous studies may partly be due to differences in the age of participants between the studies. In fact, some longitudinal studies (Bradley et al., 2001; Georgiou et al., 2021) have reported that the relationships between parent teaching of reading at home and children’s early reading skills may change depending on children’s developmental phases.

Several studies have also suggested that the relationship between HLE and children’s literacy skills may be reciprocal and that children’s early literacy skills can impact parental involvement in home literacy activities (Sénéchal and LeFevre, 2014; Silinskas et al., 2021). More specifically, previous studies have indicated that PT often shows positive associations with children’s literacy skills in kindergarten, while the relationship may become negative after formal instruction commences at primary school (Silinskas et al., 2010b; Deng et al., 2015; Georgiou et al., 2021; Silinskas et al., 2021). Interestingly, this developmental shift in the relationship between parent teaching and children’s literacy skills has been reported to be slightly delayed among English-speaking families (Georgiou et al., 2021) compared to those in relatively transparent orthographies (Silinskas et al., 2012; Inoue et al., 2018a), possibly reflecting the nature of the orthography and parents’ perceptions for their children’s reading achievement. Therefore, the association of children’s performances with PT may be partly different depending on the script children are learning to read, the educational system, and their developmental phases.

Beyond HLE, some researchers have argued that parental expectations and emotions toward children’s achievement could be a driving factor in the effect of children’s early literacy performance on later HLE (Pomerantz and Eaton, 2001; Silinskas et al., 2010a, 2013). For example, parental negative affect, such as irritation and frustration, feeling stressed, or worry about their children’s performance, may result in more controlling parental practices (Pomerantz and Eaton, 2001). Similarly, parents’ expectations for their child’s future performance may reflect the child’s current performance levels and trigger certain literacy-related activities at home (Lynch et al., 2006; Froiland et al., 2012; Liu et al., 2018; Zhang et al., 2020). Indeed, Silinskas et al. (2015) found that children’s performance in Grade 1 had a negative effect on mothers’ negative affect, help, and monitoring. In addition, they found that the lower the children’s academic performance from Grade 1 to Grade 4, the more homework assistance mothers provided. Liu et al. (2018) also showed that parents’ expectation was associated with both parent teaching and children’s word reading skills in kindergarten. Moreover, Froiland et al. (2012) reported that parents’ expectation in kindergarten was associated with home literacy support, and that it exerted a positive effect on adolescent academic achievement *via* expectations held in Grade 8.

### 1.2. Japanese Hiragana and Kanji writing system

The Japanese writing system uses two contrastive types of scripts in combination: Kana (Hiragana and Katakana) and Kanji (Koda, 2017). Of the two types of Kana scripts, Japanese children usually learn Hiragana first. Hiragana is a transparent orthography in which each character represents a syllable or mora (a syllable-like phonological unit), and the correspondences between characters and sounds are highly consistent. For example, the Hiragana character お represents the same mora /o/ across different words, such asおや /o-ya/ ‘parent,’ かお /ka-o/ ‘face,’ and おもしろい /o-mo-shi-ro-i/ ‘interesting.’ In contrast, Kanji is a morphographic script originating from Chinese in which one character can represent multiple morphemic units that are frequently multisyllabic (e.g., 生 can mean ‘birth’, ‘life’, and ‘raw’, and it can be read as /sei/, /syo/, /sei/, /u/, and /nama/ depending on the word context). Most Kanji characters correspond to two types of phonological representations: the original Chinese pronunciation (*On*-reading), which is mainly used for compound words, and the Japanese translation of the original Chinese character (*Kun*-reading), which is more common for single-character words. On-readings are generally single syllables, and thus, Kanji characters can be considered morphosyllabic when they are read in On-readings. Japanese children receive formal instruction in Hiragana at the beginning of primary school and start learning Kanji characters in the middle of the first grade with frequent characters (e.g., 花 /hana/ ‘flower’, 男 /otoko/ ‘man’). As children advance through grades, they learn characters with increasingly abstract meanings and complex forms (e.g., 難 /nan/ ‘difficulty’). Children’s early texts are frequently written only in Hiragana (e.g., かわであそぶ /ka-wa-de-a-so-bu/ ‘play in a river’), but the rate of Kanji use in texts gradually increases as children learn more Kanji characters (e.g., 川で遊ぶ /kawa-de-aso-bu/).

### 1.3. Home literacy environment and early literacy skills in Japanese

To our knowledge, only a few studies in Japanese have examined the longitudinal links between HLE and children’s reading skills during the transition period from kindergarten to early primary grades (Hamano and Uchida, 2012; Inomata et al., 2016; Inoue et al., 2018a), and their findings were mixed. Inomata et al. (2016) showed that parent teaching was associated with spelling in Hiragana in 5-to 6-year-old Japanese kindergarteners. In addition, they reported that neither shared book reading, nor parent teaching was associated with reading in Hiragana, after controlling for children’s cognitive-linguistic skills (phonological processing, rapid naming, visual processing, and receptive vocabulary). Hamano and Uchida (2012) reported that the number of books at home, in addition to parents’ education level and family income, was associated with Hiragana literacy skills in 3-to 5-year-old Japanese children. In contrast, Inoue et al. (2018a) found that children’s Hiragana reading skills in Grade 1 were negatively associated with parent teaching in Grade 2, suggesting that Japanese parents adjusted their involvement to their child’s literacy skills. Indeed, given that over 90% of third-year kindergarten children in Japan can master reading basic Hiragana characters before formal literacy instruction in school (Shimamura and Mikami, 1994; Ota et al., 2018), it is natural for parents to be sensitive to their children’s performance during the transition period from kindergarten to primary school.

However, there are some important limitations in the existing HLE studies in Japanese. First, despite the unique characteristics of the Japanese writing system that requires children to learn two scripts (syllabic Hiragana and morphographic Kanji), the effects of HLE on children’s Hiragana and Kanji reading skills have not been analyzed separately. Thus, it remains unclear whether HLE can differentially influence early Hiragana and Kanji reading skills. Given the contrastive characteristics of the two scripts, it is important to examine the reciprocal relationship between HLE and children’s literacy skills in both scripts separately and simultaneously. Second, no longitudinal studies have covered the transition from kindergarten to the early primary grades. Finally, no previous studies in Japanese have included parental affects and expectations regarding children’s literacy performance, and thus, the potential roles of these parental factors in the associations between HLE and children’s literacy skills remain unclear.

### 1.4. The present study

In this study, we examined the relationship between HLE and children’s early reading skills in syllabic Hiragana and morphographic Kanji in a sample of Japanese parent–child dyads followed from kindergarten to Grade 3. The purpose of this study was two-fold: (1) to examine the longitudinal effects of HLE on Hiragana and Kanji reading in Japanese children from kindergarten to Grade 3; (2) to examine the longitudinal effects of parental expectations, parental affects, and child reading performance on PT of Hiragana and Kanji. This study is an important addition to the findings of previous studies because it provides evidence from the longitudinal associations between HLE, parent expectations and affects, and children’s reading skills in two different scripts covering the transition from kindergarten to the early primary grades.

The present study examined the following three research questions:

- Do the HLE components (i.e., PT, SBR, and ALR) have different effects on reading skills in Hiragana and Kanji? We hypothesized that ALR would be positively associated with reading skills in Hiragana and Kanji during the period, while the effects of PT and SBR on reading skills would be relatively limited (Hamano and Uchida, 2012; Inomata et al., 2016; Inoue et al., 2018a, 2020b).
- Are Japanese parents’ affects and expectations associated with HLE, especially PT of Hiragana and Kanji? We hypothesized that parents’ affect (operationalized as parents’ worry about their child’s homework in this study) would be positively associated with PT, but negatively with the child’s reading skills (Silinskas et al., 2010a, 2013, 2015). In addition, we expected that parents’ expectations for their child’s reading performance would be associated with parent teaching and the child’s reading skills (Lynch et al., 2006; Froiland et al., 2012; Liu et al., 2018; Zhang et al., 2020).
- Do children’s reading skills in Hiragana and Kanji differentially predict the frequency of parents’ teaching in each script? We hypothesized that Hiragana reading skills would negatively predict PT of Hiragana in kindergarten to Grade 1, and Kanji reading skills would also negatively predict PT of Kanji in Grade 2 to Grade 3 (Silinskas et al., 2010a, 2021; Deng et al., 2015; Inoue et al., 2018a; Georgiou et al., 2021).

## 2. Materials and methods

### 2.1. Research design

To examine the relationship between HLE and reading skills in Hiragana and Kanji at different time periods, we conducted longitudinal analyzes for Hiragana reading from kindergarten to Grade 1, and for Kanji reading from Grade 1 to Grade 3. The children were tested four times over the 3 years on word reading skills (Hiragana reading accuracy, Hiragana word reading fluency, and Kanji reading accuracy) with 10-to 12-month intervals: at the end of kindergarten (Time 1) and the middle of Grade 1 (Time 2), Grade 2 (Time 3), and Grade 3 (Time 4).

In Japan, formal literacy instruction generally begins in Grade 1. Children first learn Hiragana and then begin to learn Kanji in the middle of Grade 1. According to the national curriculum (Ministry of Education, Culture, Sports, Science and Technology, 2017), children formally learn 80 Kanji characters in Grade 1, 160 in Grade 2, and 200 in Grade 3 at school. Given this sequential learning of Hiragana and Kanji set by the national curriculum, we assessed Hiragana reading accuracy only in kindergarten, Hiragana reading fluency in kindergarten and Grade 1, and Kanji reading accuracy in Grades 1 to 3. HLE and parental expectations and affect were assessed by parent questionnaires at all four time points.

### 2.2. Participants

We approached several kindergartens and elementary schools to recruit participating children in Okayama city, Japan. The participants were Japanese kindergarten children (*N* = 83, *M*_age_ = 75.6 months, SD = 3.4) who were given parental permission to participate in this study, and they were followed until the middle of Grade 3. Using G*Power (Version 3.1.9.7; Faul et al., 2009) with an effect size of 0.2, the α error probability of 0.05, the 1-β error probability of 0.8, and the number of predictors of five (see below for details of the analysis), the required sample size for multiple regression analysis was estimated to be 70. The sample size of this study generally met these conditions. All participants were native Japanese speakers. In addition, the parents of the children participated in the study by completing a questionnaire. The median of mothers’ education attainment in our sample was “graduated from junior college or technical college level.” This was slightly higher than the general population, according to the latest census data (Ministry of Internal Affairs and Communications, 2020). Parental and school consent was obtained prior to testing. Ethical approval was obtained from Okayama University.

### 2.3. Measures

#### 2.3.1. Children’s reading skills

##### 2.3.1.1. Hiragana reading accuracy

The Hiragana nonword decoding task (Tanji and Inoue, 2022) was used. The task consisted of 15 four-character Hiragana nonwords. The nonwords included 46 basic Hiragana characters with four voiced, one semi-voiced, and five special sounds, and they were arranged in terms of increasing level of difficulty. The items were divided into three columns with five nonwords on a page. Children were required to read the nonwords as accurately as possible. The total number of correct answers was considered, and the maximum score was 15. Cronbach’s alpha reliability in our sample was 0.87.

##### 2.3.1.2. Hiragana reading fluency

The Hiragana word reading fluency task (Inoue et al., 2020a; Tanji and Inoue, 2022) was used. The task comprised 104 four-character Hiragana words taken from Grade 1 textbooks. The words were divided into four columns with 20 or 21 words on a page. A practice trial that required reading an eight-word list was conducted before testing to ensure familiarity. Children were asked to read the list of words as quickly as possible. The score was the number of words correctly read within 45 s with a maximum score of 104. The correlation between kindergarten and Grade 1 was 0.93, indicating the stability of the measure.

##### 2.3.1.3. Kanji reading accuracy

The Kanji reading task was adopted from Inoue et al. (2017) and used to assess Kanji reading accuracy. In Grades 1 and 2, the task consisted of 50 Kanji characters (10 characters from Grades 1 to 4 and 5 characters from Grades 5 and 6 were selected from the national standard curriculum; Ministry of Education, Culture, Sports, Science and Technology, 2017). In Grade 3, we used a list of 120 Kanji characters (20 characters from each grade from 1 to 6 were selected). The number of items was increased for Grade 3 children to avoid a possible ceiling effect. The items were arranged according to an increasing level of difficulty, and five characters were printed on each page. Children were required to read the Kanji characters as accurately as possible. A child’s score was the total number of correct answers. Cronbach’s alpha reliability in our sample was 0.97, 0.96, and 0.98 for Grades 1 to 3, respectively.

#### 2.3.2. Parents’ questionnaire

The parents were asked to answer questions about (a) mothers’ education level, (b) HLE (parent teaching of Hiragana and Kanji, shared book reading, and access to literacy resources), (c) parents’ needs for early literacy support by teachers, (d) parents’ expectations for children’s literacy skills, and (e) parents’ worry about children’s homework. Most of the questions were adopted from previous studies, including those in Japanese (e.g., Deng et al., 2015; Inoue et al., 2018a).

##### 2.3.2.1. Mothers’ education

We asked parents to report mothers’ highest achieved education level among six options ranging from *junior high school graduate* (0) to *completed master’s course at graduate school* (5).

##### 2.3.2.2. Parent teaching in Hiragana

PT in Hiragana was assessed using three 5-point Likert scale questions. The first question asked “how often did parents teach their child to read Hiragana characters,” and parents responded on a scale ranging from *never* (0) to *daily* (4). The second and third questions asked “how often did parents teach their child to read Hiragana words” and “how often did parents teach their child to write Hiragana characters.” PT in Hiragana was assessed in kindergarten and Grade 1. Cronbach’s alpha reliability in our sample was 0.85 in kindergarten and 0.90 in Grade 1.

##### 2.3.2.3. Shared book reading

SBR was assessed using two 5-point Likert scale questions. The first question asked “how often did parents read to their child on weekdays (Monday to Friday),” and parents responded on a scale ranging from *never* (0) to *daily* (4). The second question asked “how often did parents read to their child on weekends (Saturday and Sunday),” and parents responded on the same scale. SBR was assessed four times in kindergarten, Grades 1, 2, and 3. In our sample, Cronbach’s alpha reliability was 0.69, 0.91, 0.91, and 0.88 for kindergarten and Grades 1 to 3, respectively.

##### 2.3.2.4. Parent teaching in Kanji

PT in Kanji was assessed using two 5-point Likert scale questions. The first question asked “how often did parents teach their child to read Kanji characters,” and parents responded on a scale ranging from *never* (0) to *daily* (4). The second question asked “how often did parents teach their child to write Kanji characters.” PT in Kanji was assessed three times in Grades 1, 2, and 3. Cronbach’s alpha reliability in our sample was 0.90, 0.91, and 0.85 for Grades 1 to 3, respectively.

##### 2.3.2.5. Access to literacy resources

We used a 5-point Likert scale question that asked “how often did parents go to the library or bookstore with their child,” and parents responded on a scale ranging from *non*e (0) to *two to 3 days in a week* (4). ALR was assessed three times in kindergarten, Grade 2, and Grade 3. The correlations between time points ranged from 0.43 to 0.57.

##### 2.3.2.6. Parents’ needs for early literacy support by teachers

Two 5-point Likert scale questions were used to assess parents’ needs for early literacy support by teachers. The first question asked “whether parents wanted their child’s teacher to assess the child’s literacy skills as soon as he/she goes to primary school,” and parents responded on a scale ranging from *strongly disagree* (0) to *strongly agree* (4). The second question asked “whether parents wanted their child’s teacher to provide early literacy support when the child goes to primary school,” and parents responded on the same scale. Parents’ needs were only assessed in kindergarten. Cronbach’s alpha reliability in our sample was 0.91.

##### 2.3.2.7. Parents’ expectations for child’s literacy skills

Parents were asked to report on their expectations about their child’s current and future literacy skills using two 5-point Likert scale questions. The questions asked “how well did parents think their child was/would be doing in reading and writing at the time/in the future,” and parents responded on a scale ranging from *not good at all* (0) to *very well* (4). Parents’ expectations were assessed three times in Grades 1, 2, and 3. Cronbach’s alpha reliability in our sample was 0.79, 0.90, and 0.93 for Grades 1 to 3, respectively.

##### 2.3.2.8. Parents’ worry about child’s homework

Parents were asked to report on their worries about their child’s homework using a 5-point Likert scale question. The question asked “how much trouble did parents have with their child’s homework,” and parents responded on a scale ranging from *strongly disagree* (0) to *strongly agree* (4). Parents’ worry about homework was assessed three times in Grades 1, 2, and 3. The correlations between time points ranged from 0.56 to 0.72.

### 2.4. Procedure

The children were assessed at the end of kindergarten (January/February) and the middle of Grades 1, 2, and 3 (September/October): Time 1 to Time 4, respectively. Kanji reading accuracy was not assessed in kindergarten because children had not started learning Kanji characters and, thus, a floor effect was expected. In addition, Hiragana reading accuracy was not assessed in Grades 1 to 3 because a ceiling effect was expected (see Mikami et al., 2008; Ota et al., 2018). The children were tested individually in their kindergartens and schools during school hours by trained experimenters. Administration and scoring were standardized across all children by using a manual of test administration procedures and scoring sheets to enhance the reliability of our data. Several experimenters visited the kindergartens/schools and tested children simultaneously. The testing time lasted for 30 min at Time 1 and 20 min at Time 2 to Time 4. The data collection schedule at the kindergartens/schools was developed in consultation with the school principal and the participants’ teachers in each kindergarten/school prior to the data collection at each time point. The parents completed the questionnaires at approximately the same time as their children. The distribution and collection of the questionnaires were assisted by the participants’ teachers.

### 2.5. Statistical analysis

To examine the longitudinal associations between the HLE components, parents’ affects, and early reading skills in Hiragana and Kanji, we performed multiple regression analyzes using SPSS 25.0 (IBM Corp, 2017). To avoid alpha inflation due to a large number of hypothesis testing using the same variables, we focused on longitudinal associations between the variables and did not test concurrent associations. Separate sets of regression models were estimated for the two scripts. First, two models were estimated for predicting children’s Hiragana reading and PT in Hiragana, respectively. In Model 1, to examine the effect of HLE on Hiragana reading, the three HLE components (PT, SBR, and ALR) in kindergarten were entered for predicting Hiragana reading fluency in Grade 1. In Model 2, to examine the effect of parental affect and child’s reading skill on PT in Hiragana, mother’s education, parents’ needs for early literacy support by teachers, and Hiragana reading fluency in kindergarten were entered for predicting PT in Hiragana in Grade 1. In addition, to examine the unique effect of the predictor variables, the models were estimated both with and without controlling for the effect of autoregressors (i.e., the same variables at the previous time point). Next, four separate regression models were estimated for predicting Kanji reading accuracy and PT in Kanji. In Model 1, to examine the effect of HLE on Kanji reading, parents’ expectations, PT in Kanji, and SBR in Grade 1 were entered for predicting Kanji reading accuracy in Grade 2. In Model 2, to examine the effect of parental affects and expectations and child’s reading skill on PT in Kanji, mother’s education, parents’ expectations, parents’ worry, and Kanji reading accuracy in Grade 1 were entered for predicting PT in Kanji in Grade 2. In Model 3, parents’ expectations and the three HLE components in Grade 2 were entered for predicting Kanji reading accuracy in Grade 3. Finally, in Model 4, mother’s education, parents’ expectations, parents’ worry, and Kanji reading accuracy in Grade 2 were entered for predicting PT in Kanji in Grade 3. All four models were estimated both with and without controlling for the effects of autoregressors to examine the unique effect of the predictor variables.

## 3. Results

### 3.1. Preliminary data analysis

Table 1 displays the descriptive statistics for all measures. The distributional properties of the variables indicated that Kanji reading accuracy was positively skewed at Time 2. Therefore, square root transformation was applied to improve the distribution. In addition, Hiragana reading accuracy at Time 1 was negatively skewed, and reflect and square root transformation was performed. The reflected scores were multiplied by −1 to correct for direction. In addition, outliers on several measures in each sample (defined as over 2.5 *SD* above/below the mean) were winsorized to the next non-outliers’ score of ±1 to reduce their potential effects on the results (Tabachnick and Fidell, 2013).

**Table 1:** Descriptive statistics for child and parent measures.

	Time 1				Time 2				Time 3				Time 4			
	Kindergarten (*N* = 83)				Grade 1 (*N* = 79)				Grade 2 (*N* = 69)				Grade 3 (*N* = 59)			
Measure (max)	M	SD	Skew	Kurt	M	SD	Skew	Kurt	M	SD	Skew	Kurt	M	SD	Skew	Kurt
Child measures																
Age in months	75.59	3.36	−0.11	−0.99	83.67	3.48	−0.09	−1.07	96.10	3.54	−0.16	−1.06	108.14	3.39	−0.10	−1.07
Hiragana reading accuracy (15)	11.96	3.07	−1.73	3.69	–	–	–	–	–	–	–	–	–	–	–	–
Hiragana reading fluency (104)	27.71	14.31	−0.36	−0.21	40.46	15.14	0.16	−0.34	–	–	–	–	–	–	–	–
Kanji reading accuracy (Grade 1-Grade 2;50, Grade 3;120)	–	–	–	–	8.49	8.86	1.89	3.85	22.13	9.69	1.18	0.65	57.36	22.63	0.72	−0.24
Parent measures																
Mother’s education (6)	3.80	1.27	−0.43	−1.25	–	–	–	–	–	–	–	–	–	–	–	–
Parent teaching (PT)																
Teach to read Hiragana characters (4)	2.40	0.94	−0.89	0.47	1.99	1.33	0.13	−1.26	–	–	–	–	–	–	–	–
Teach to read Hiragana words (4)	2.48	0.91	−0.33	−0.25	2.16	1.20	0.06	−1.04	–	–	–	–	–	–	–	–
Teach to write Hiragana characters (4)	2.32	0.95	−0.69	2.00	2.45	1.20	−0.34	−0.93	–	–	–	–	–	–	–	–
Shared book reading (SBR)																
Read to child: weekdays (4)	2.57	1.14	−0.33	−0.92	2.13	1.15	0.22	−1.02	1.23	1.23	0.51	−0.95	0.92	1.10	1.05	0.39
Read to child: weekend (4)	3.00	1.14	−0.96	−0.10	2.44	1.26	−0.33	−1.07	1.26	1.24	0.63	−0.62	1.29	1.33	0.36	−1.38
Parent teaching in Kanji (PTK)																
Teach to read Kanji characters (4)	–	–	–	–	2.18	1.21	−0.27	−0.91	2.59	0.93	−1.11	1.27	2.12	1.00	−0.78	−0.14
Teach to write Kanji characters (4)	–	–	–	–	1.60	1.37	0.36	−1.11	2.42	1.05	−0.85	0.18	1.90	1.11	−0.19	−0.72
Access to literacy resources (ALR)																
Go to bookstores/libraries (4)	1.71	0.67	0.16	−0.41	–	–	–	–	1.58	0.73	0.33	−0.38	1.54	0.65	0.03	−0.16
Parents’ needs for early literacy support by teachers																
Needs for teachers to understand child’s reading skill (4)	2.72	1.07	−0.43	−0.78	–	–	–	–	–	–	–	–	–	–	–	–
Needs for early intervention in reading skill by teachers (4)	2.63	1.10	−0.58	−0.36	–	–	–	–	–	–	–	–	–	–	–	–
Parents’ expectations for child’s literacy skills																
Expectation for child’s reading skills: now (4)	–	–	–	–	2.17	0.89	0.14	−0.88	2.38	1.05	0.08	−1.18	2.25	0.90	0.34	−0.56
Expectation for child’s reading skills: future (4)	–	–	–	–	2.71	0.83	−0.58	−0.02	2.68	0.93	−0.26	−0.73	2.53	0.97	0.04	−0.94
Parents’ worry about child’s homework																
Worry about child’s homework engagement (4)	–	–	–	–	1.59	1.07	0.35	−0.61	1.41	1.08	0.85	0.50	1.51	1.06	0.75	0.26

### 3.2. Correlation analysis

Table 2 presents the correlations between the HLE components and parents’ variables across time points. The results showed that parents’ expectations, parents’ needs for teachers’ support, and parents’ worry for children’s performance were correlated with PT. Specifically, parents’ needs for teachers’ support were positively correlated with PT in Hiragana in kindergarten (*r* = 0.33), indicating that the more parents wanted early support, the more often they taught Hiragana literacy skills to their children. In Grade 1, parents’ expectations were negatively correlated with PT in Hiragana (*r* = −0.37), and parents’ worry was positively correlated with PT in Hiragana (*r* = 0.42). Similarly, parents’ expectations were negatively correlated with PT in Kanji (*r* = −0.34), and parents’ worry was positively correlated with PT in Kanji in Grade 2 (*r* = 0.43). Furthermore, parents’ worry, but not their expectations, was positively correlated with PT in Kanji in Grade 3 (*r* = 0.58).

**Table 2:** Correlation between parent variables.

		1	2	3	4	5	6	7	8	9	10	11	12	13	14	15	16	17	18	19	20
1	MotherEduc	–	–	–	–	–	–	–	–	–	–	–	–	–	–	–	–	–	–	–	–
2	PTH_K	−0.12	–	–	–	–	–	–	–	–	–	–	–	–	–	–	–	–	–	–	–
3	SBR_K	0.15	0.29*	–	–	–	–	–	–	–	–	–	–	–	–	–	–	–	–	–	–
4	ALR_K	0.19	0.12	0.36**	–	–	–	–	–	–	–	–	–	–	–	–	–	–	–	–	–
5	PNeeds_K	−0.23*	0.33**	0.01	0.01	–	–	–	–	–	–	–	–	–	–	–	–	–	–	–	–
6	PTH_G1	−0.15	0.12	0.07	−0.09	0.16	–	–	–	–	–	–	–	–	–	–	–	–	–	–	–
7	PTK_G1	−0.08	0.28*	0.09	−0.03	0.19	0.35**	–	–	–	–	–	–	–	–	–	–	–	–	–	–
8	SBR_G1	0.20	0.26*	0.74***	0.35**	0.04	0.12	0.09	–	–	–	–	–	–	–	–	–	–	–	–	–
9	PExpect _G1	0.22	0.26*	0.13	0.07	−0.12	−0.37**	0.07	0.07	–	–	–	–	–	–	–	–	–	–	–	–
10	PWorry_G1	−0.07	−0.03	−0.01	−0.18	0.24*	0.42***	0.06	−0.02	−0.49***	–	–	–	–	–	–	–	–	–	–	–
11	PTK_G2	0.10	−0.03	0.04	−0.05	0.24	37**	0.29*	0.21	−0.25*	0.31*	–	–	–	–	–	–	–	–	–	–
12	SBR_G2	0.20	0.29*	0.58***	0.17	−0.02	0.00	0.12	0.78***	0.11	0.07	0.15	–	–	–	–	–	–	–	–	–
13	PExpect_G2	0.07	0.19	0.30*	0.15	−0.19	−0.48***	0.01	0.14	0.67***	−0.48***	−0.34**	0.13	–	–	–	–	–	–	–	–
14	ALR_G2	0.02	−0.03	0.18	0.56***	0.04	−0.08	0.19	0.15	−0.08	−0.14	0.02	0.05	0.06	–	–	–	–	–	–	–
15	PWorry_G2	0.04	0.01	−0.05	−0.09	0.34*	0.43***	0.10	0.05	−0.45***	0.56***	0.43***	0.12	−0.43***	−0.07	–	–	–	–	–	–
16	PTK_G3	0.19	0.36**	0.02	0.17	0.20	0.39**	0.26*	0.25	0.00	0.19	0.38**	0.12	−0.12	0.00	0.38**	–	–	–	–	–
17	SBR_G3	0.06	0.29*	0.56***	0.19	0.21	0.10	0.30*	0.56***	0.21	−0.01	0.25	0.60***	0.08	0.01	0.23	0.38**	–	–	–	–
18	PExpect_G3	0.16	0.17	0.27*	0.34**	−0.15	−0.38**	0.14	0.17	0.62***	−0.44***	−0.26	0.10	0.74***	0.15	−0.38**	0.02	0.17	–	–	–
19	ALR_G3	−0.05	0.21	0.05	0.42**	0.11	0.09	0.29*	0.11	−0.07	−0.19	0.04	0.03	−0.15	0.57***	0.09	0.11	0.14	0.05	–	–
20	PWorry_G3	−0.04	0.08	−0.13	−0.09	0.38**	0.49***	0.04	0.07	−0.45***	0.57***	0.46***	−0.02	−0.37**	−0.06	0.72***	0.58***	0.28*	−0.27*	0.05	–

MotherEduc = mother’s education, PTH = parent teaching in Hiragana, SBR = shared book reading, PExpect = parents’ expectations for child’s reading skills, ALR = access to literacy resources, PTK = parent teaching in Kanji, PNeeds = parents’ needs for early literacy support by teachers, PWorry = parents’ worry about child’s homework, K = Kindergarten, G1 = Grade 1, G2 = Grade 2, G3 = Grade 3.

*p < 0.05; **p < 0.01; ***p < 0.001.

Table 3 presents the correlations between children’s and parents’ variables across time points. Neither PT in Hiragana nor SBR in kindergarten was correlated with Hiragana and Kanji reading skills at any time point. In contrast, PT in Hiragana in Grade 1 was negatively correlated with Hiragana reading fluency in kindergarten and Grade 1 (*r*s = −0.52 and − 0.49, respectively). In contrast, ALR in kindergarten was positively correlated with Hiragana reading fluency in Grade 1 (*r* = 0.35), and ALR in Grades 2 and 3 was correlated with Kanji reading accuracy in Grades 2 and 3 (*r*s = 0.33 and 0.29, respectively). In addition, parents’ expectations in Grade 1 were correlated with Kanji reading in Grade 1 (*r* = 0.31), and parents’ expectations in Grade 2 were correlated with Kanji reading in Grades 2 and 3 (*r*s = 0.31 and 0.46, respectively). On the other hand, parents’ worry in Grade 1 was negatively correlated with Hiragana reading fluency (*r* = −0.47) and Kanji reading (*r* = −0.38) at the same time point, and parents’ worry in Grades 2 and 3 was also negatively correlated with Kanji reading in Grades 2 and 3 (*r*s = −0.28 and − 0.31, respectively).

**Table 3:** Correlation between child and parent variables.

Child variables	Parent variables										
	*K*					G1					
	PT in Hiragana	SBR	ALR	Needs		PT in Hiragana	PT in Kanji	SBR	Expectations	Worry	
*K*											
Hiragana reading accuracy	−0.04	−0.15	0.18	−0.22		−0.35**	−0.24*	−0.11	0.23	−0.20	
Hiragana reading fluency	0.04	0.16	0.35**	−0.29*		−0.52***	−0.07	−0.02	0.41***	−0.41***	
G1											
Hiragana reading fluency	0.01	0.14	0.35**	−0.33**		−0.49***	0.00	−0.01	0.39**	−0.47***	
Kanji reading accuracy	−0.00	0.06	0.31**	−0.08		−0.42***	0.14	−0.10	0.31**	−0.38**	
G2											
Kanji reading accuracy	0.03	0.08	0.36**	−0.29*		−0.31**	0.15	−0.13	0.21	−0.34**	
G3											
Kanji reading accuracy	−0.06	0.17	0.46***	−0.25		−0.40**	0.09	−0.10	0.24	−0.41***	
**Child variables**	**Parent variables**										
	**G2**					**G3**					
	**PT in Kanji**	**SBR**	**ALR**	**Expectations**	**Worry**	**PT in Kanji**	**SBR**	**ALR**	**Expectations**	**Worry**	**Mother’s education**
*K*											
Hiragana reading accuracy	−0.01	0.05	0.19	0.20	−0.12	0.13	−0.04	0.14	0.19	−0.02	0.16
Hiragana reading fluency	−0.20	0.13	0.29*	0.45***	−0.38**	−0.05	0.06	0.33**	0.55***	−0.35**	0.16
G1											
Hiragana reading fluency	−0.13	0.10	0.30*	0.43***	−0.33**	−0.08	0.03	0.28*	0.56***	−0.36**	0.15
Kanji reading accuracy	−0.15	0.09	0.23	0.36**	−0.29*	−0.05	−0.02	0.20	0.52***	−0.28*	0.14
G2											
Kanji reading accuracy	−0.17	0.03	0.33**	0.31**	−0.28*	−0.04	−0.09	0.36**	0.38**	−0.31*	0.12
G3											
Kanji reading accuracy	−0.12	−0.01	0.40**	0.29*	−0.30*	−0.09	−0.06	0.29*	0.46***	−0.31*	0.09

PT = parent teaching, SBR = shared book reading, ALR = access to literacy resources, Needs = parents’ needs for early literacy support by teachers, Expectations = parents’ expectations for child’s reading skills, Worry = parents’ worry about child’s homework, K = Kindergarten, G1 = Grade 1, G2 = Grade 2, G3 = Grade 3.

*p < 0.05; **p < 0.01; ***p < 0.001.

### 3.3. Multiple regression analysis

Table 4 shows the results of multiple regression analyzes for predicting Hiragana reading skills and PT in Hiragana in Grade 1. In Model 1, whereas neither PT in Hiragana nor SBR predicted Hiragana reading fluency in Grade 1, ALR predicted Hiragana reading fluency in Grade 1 (*β* = 0.30). However, ALR did not hold a unique effect on Hiragana reading fluency in Grade 1 when the effect of Hiragana reading fluency in kindergarten was controlled (see Model 1b). In contrast, Model 2 showed that Hiragana reading fluency in kindergarten negatively predicted PT in Hiragana in Grade 1 (*β*s = −0.60 to −0.64) after controlling for the effect of PT in Hiragana in kindergarten, indicating that PT in Hiragana in Grade 1 was more frequent when their child’s early Hiragana reading was poor.

**Table 4:** Multiple regression analyzes for HLE and Hiragana reading from kindergarten to grade 1.

Predictors	*β*	Total *R^2^*	Total adjusted *R^2^*
Model 1: Prediction of Hiragana reading fluency in G1			
1a: Without autoregressor			
Shared book reading (SBR)_K	0.02		
Access to literacy resource (ALR)_K	0.30*		
Parent teaching (PT) in Hiragana_K	0.01	0.09	0.05
1b: With autoregressor			
Shared book reading (SBR)_K	0.02		
Access to literacy resource (ALR)_K	−0.05		
Parent teaching (PT) in Hiragana_K	0.01		
Hiragana reading fluency in K	0.93***	0.83	0.82
Model 2: Prediction of parent teaching in Hiragana in G1			
2a: Without autoregressor			
Mother’s education	0.03		
Parents’ needs for early literacy support by teachers_K	−0.06		
Hiragana reading fluency_K	−0.60***	0.33	0.30
2b: With autoregressor			
Mother’s education	0.05		
Parents’ needs for early literacy support by teachers_K	−0.13		
Hiragana reading fluency_K	−0.64***		
Parent teaching (PT) in Hiragana_K	0.20^†^	0.37	0.33

K = Kindergarten, G1 = Grade 1.

*p < 0.05, **p < 0.01, ***p < 0.001, ^†^*p* = 0.07.

Table 5 shows the results of multiple regression analyzes for predicting Kanji reading accuracy and PT in Kanji in Grades 2 and 3. Model 1 showed that none of the parents’ variables in Grade 1 predicted Kanji reading accuracy in Grade 2. Similarly, Model 2 showed that Kanji reading accuracy in Grade 1 did not predict PT in Kanji in Grade 2. In contrast, in Model 3, ALR in Grade 2, but not PT in Kanji and SBR, predicted Kanji reading in Grade 3 (*β* = 0.42). However, ALR in Grade 2 did not hold a unique effect on Kanji reading accuracy in Grade 3 when the effect of autoregressor was controlled (see Model 3b). Finally, Model 4 showed that parents’ worry about their child’s homework in Grade 2 was associated with PT in Kanji in Grade 3 (*β* = 0.30), indicating that the more concerned parents were about their child’s homework, the more often they taught Kanji literacy skills to their children. However, the effect of parents’ worry in Grade 2 on PT in Kanji in Grade 3 became nonsignificant when the effect of PT in Kanji in Grade 2 was taken into account (see Model 4b).

**Table 5:** Multiple regression analyzes for HLE and Kanji reading from grade 1 to grade 3.

Predictors	*β*	Total *R^2^*	Total adjusted *R^2^*
HLE and Kanji reading in G1 and G2			
Model 1: Prediction of Kanji reading accuracy in G2			
1a: Without autoregressor			
Parents’ expectations for child’s literacy skills_G1	0.23		
Shared book reading (SBR)_G1	−0.13		
Parent teaching (PT) in Kanji_G1	0.13	0.08	0.03
1b: With autoregressor			
Parents’ expectations for child’s literacy skills_G1	−0.07		
Shared book reading (SBR)_G1	0.01		
Parent teaching (PT) in Kanji_G1	−0.07		
Kanji reading accuracy_G1	0.85***	0.66	0.64
Model 2: Prediction of parent teaching in Kanji in G2			
2a: Without autoregressor			
Mother’s education	0.21		
Parents’ expectations for child’s literacy skills_G1	−0.26		
Parents’ worry about child’s homework_G1	0.03		
Kanji reading accuracy_G1	−0.13	0.15	0.09
2b: With autoregressor			
Mother’s education	0.23		
Parents’ expectations for child’s literacy skills_G1	−0.27		
Parents’ worry about child’s homework_G1	−0.01		
Kanji reading accuracy_G1	−0.23		
Parent teaching (PT) in Kanji_G1	0.40**	0.30	0.24
HLE and Kanji reading in G2 and G3			
Model 3: Prediction of Kanji reading accuracy in G3			
3a: Without autoregressor			
Parents’ expectations for child’s literacy skills_G2	0.22		
Shared book reading (SBR)_G2	−0.09		
Access to literacy resources (ALR)_G2	0.42**		
Parent teaching (PT) in Kanji_G2	−0.04	0.23	0.17
3b: With autoregressor			
Parents’ expectations for child’s literacy skills_G2	−0.02		
Shared book reading (SBR)_G2	−0.05		
Access to literacy resources (ALR)_G2	0.09		
Parent teaching (PT) in Kanji_G2	−0.02		
Kanji reading accuracy_G2	0.89***	0.86	0.84
Model 4: Prediction of parent teaching in Kanji in G3			
4a: Without autoregressor			
Mother’s education	0.20		
Parents’ expectations for child’s literacy skills_G2	−0.06		
Parents’ worry about child’s homework_G2	0.30*		
Kanji reading accuracy_G2	−0.03	0.15	0.08
4b: With autoregressor			
Mother’s education	0.13		
Parents’ expectations for child’s literacy skills_G2	0.00		
Parents’ worry about child’s homework_G2	0.23		
Kanji reading accuracy_G2	−0.02		
Parent teaching (PT) in Kanji_G2	0.32*	0.24	0.16

G1 = Grade 1, G2 = Grade 2, G3 = Grade 3.

*p < 0.05, **p < 0.01, ***p < 0.001.

## 4. Discussion

This study examined the reciprocal associations between HLE and children’s reading skills in Hiragana and Kanji in a sample of Japanese parent–child dyads from kindergarten to Grade 3. Specifically, we sought to answer the three research questions: (a) Do the HLE components (i.e., PT, SBR, and ALR) have different effects on reading skills in Hiragana and Kanji, (b) are parents’ affects and expectations associated with HLE, and (c) do children’s reading skills in Hiragana and Kanji differentially predict the frequency of parents’ teaching in each script?

### 4.1. HLE and early Hiragana and Kanji reading skills

Regarding our first research question, the results showed that neither PT nor SBR predicted reading skills in Hiragana and Kanji. These results, together with similar findings from previous studies in different languages (e.g., Deng et al., 2015; Inoue et al., 2018a,b; Silinskas et al., 2021), suggest that the effect of PT and SBR may be time-sensitive and reduced as children exposed to formal literacy instruction. According to the Home Literacy Model (Sénéchal and LeFevre, 2002; Sénéchal, 2006), code-related (formal) activities (i.e., teaching of letters and words) have been found to predict word reading through its effects on letter knowledge, and meaning-related (informal) activities (i.e., shared book reading) have been to predict word reading through its effects on vocabulary and phonological awareness (e.g., Sénéchal, 2006; Silinskas et al., 2010b; Sénéchal and LeFevre, 2014; Torppa et al., 2022; Zhang et al., 2023). In Japan, it is possible that the effects of PT be limited, especially after schooling, because most children acquire letter knowledge before schooling. In addition, previous Japanese studies did not ask separate questions on the frequency of teaching Hiragana and Kanji reading (e.g., Inoue et al., 2018a; Inoue et al., 2023). Our results showed that there was no significant relationship between Hiragana and Kanji reading and each parent teaching after entering school even when the questions were separated for each script.

In contrast, ALR positively predicted reading skills in both scripts. Similar findings have been reported in several studies showing that ALR was associated with children’s reading skills (Hamano and Uchida, 2012; Inoue et al., 2018b, 2020b; Georgiou et al., 2021). Several studies in Chinese have also provided evidence for the associations between ALR and Chinese character reading (Liu et al., 2018; Zhang et al., 2020, 2023). Given these empirical findings, one interpretation of our results is that having more reading materials available in the home can provide children with opportunities to practice reading Hiragana words and contribute to learning Kanji characters, possibly partly through enriching vocabulary knowledge (Zhang et al., 2020). Another interpretation may be that having access to printed materials at home can influence children’s autonomy to read books and active interest in learning new words compared to engaging in parent-led activities such as shared book reading (see Van Bergen et al., 2017; Inoue et al., 2020b; Georgiou et al., 2021, for relevant discussions). It should be noted, however, that although ALR was consistently and weakly to moderately correlated with children’s reading skills across grades (Table 3), it did not exert a unique effect on reading outcomes in either script when the effect of autoregressors was controlled. Therefore, caution should be exercised when interpreting our findings; while ALR and children’s word reading skills may be associated, the effect of ALR further development of reading skills may be relatively limited.

### 4.2. Parental affects and expectations and parental teaching

Regarding the associations of parents’ affects and expectations with HLE, parents’ worry about their child’s homework in Grade 2 was positively associated with PT in Kanji in Grade 3. In contrast, parents’ expectations did not predict PT in Kanji in any grade (see Model 4 in Table 5). Similar findings have been reported in several previous studies (Pomerantz and Eaton, 2001; Silinskas et al., 2015). For example, Silinskas et al. (2015) showed that children’s academic performance in Grade 1 predicted their mother’s practices (e.g., helping and monitoring their child) in homework in Grade 3 through its effects on parental negative affect (e.g., feeling hopeless, frustrated) on homework. Pomerantz and Eaton (2001) also showed that parents’ concern for their children’s academics has a positive effect on parental practices (e.g., monitoring and helping with homework) among parents with children in Grades 4 to 6. Taken together, these results suggest that negative parental affect, such as their worry about children’s homework, may be more closely associated with parental involvement in children’s learning at home than parents’ expectations for their child’s literacy skills. However, it should be noted that although parents’ worry was weakly to moderately correlated with parent teaching in Kanji in Grades 2 and 3, it did not exert a unique effect on parent teaching in Kanji when the effect of autoregressor was controlled (see Model 4 in Table 5), suggesting that its effect on parent teaching might be relatively weak.

### 4.3. Early word reading skills and parental teaching

The results further showed that while Hiragana reading in kindergarten was not correlated with PT in Hiragana in kindergarten (Table 3), it negatively predicted PT in Hiragana in Grade 1 even after controlling for PT in Hiragana in kindergarten (Table 4). The negative relationship aligned with previous findings in different cultural contexts, including Japanese (Manolitsis et al., 2011; Deng et al., 2015; Inoue et al., 2018a; Silinskas et al., 2021). Specifically, Inoue et al. (2018a) showed that Hiragana reading in Grade 1 negatively predicted parent teaching of letters and words in Grade 2. Our results suggested that the negative relationship might be relatively stronger during the transition from kindergarten to Grade 1. Similar to previous studies in other cultures (e.g., Silinskas et al., 2010b, 2013, 2021), we found that children’s reading skills have a negative effect on PT at times before and after the start of formal instruction in Japan, indicating that in the early years of schooling, the more parents have children with low reading levels, the more likely they are to be involved in direct teaching. Silinskas et al. (2021) suggested that parents might adjust their frequency of teaching to their children’s needs for support following the transition to Grade 1. In this study, parents’ needs for children’s literacy support was associated positively with PT in Hiragana in kindergarten (*r* = 0.33, see Table 2) and negatively with Hiragana reading fluency in kindergarten (*r* = −0.29, see Table 3). These results support the notion that parents’ affects and expectations for their children’s performance can be reflected in parents’ involvement at home, such as PT (Deng et al., 2015; Hemmerechts et al., 2016; Georgiou et al., 2021). Parents may be particularly responsive to their children’s literacy skills at the beginning of formal schooling in Grade 1.

In contrast, children’s Kanji reading in Grades 1 and 2 did not uniquely predict PT in Kanji in Grades 2 and 3. This might be because parents adjusted the frequency of Kanji teaching according to their child’s autonomy in doing their homework rather than the performance level of the child’s reading (Silinskas et al., 2015). It is also possible that the nature of parental involvement in children’s learning may have changed from Grade 1 onwards in response to formal Kanji literacy instruction in primary school. Specifically, once formal instruction of Kanji commences, Japanese parents may be less likely to teach Kanji directly at home and spend more time on monitoring and helping with their children’s homework (see Table 1).

### 4.4. Implications

Our findings have some important educational implications. First, ALR at home may be associated with early literacy development in both Hiragana and Kanji in Japanese. This implies that activities in which children can engage in self-regulated and playful activities may have a greater impact on their learning than parent-initiated activities (e.g., Grolnick and Ryan, 1987, 1989). In addition, a meta-analytic review (Sénéchal and Young, 2008) showed that parents listening to their children read could play a facilitative role in children’s reading acquisition. We should encourage educators and parents not only to teach literacy to their children directly but also to consider providing more access to printed materials at home to enhance children’s autonomy in accessing written material that can create a foundation for future literacy development. Moreover, the association between children’s early reading skills and later parents’ involvement suggests that educators should increase their communication with parents regarding their child’s literacy performance and how they can help them achieve their learning goals.

### 4.5. Limitations

Several limitations of our study are worth noting. First, our sample size was relatively small. Thus, caution is required with interpreting the results. Consequently, the findings should be replicated in future studies with a larger and more representative sample. Second, we used single-item measures to assess ALR and parents’ affect. This may have caused a potential underestimation of their effects on reading skills and HLE. Third, we did not assess parental reading levels or reading history. Some researchers have suggested that parents’ reading proficiency may be associated with both HLE and children’s reading development (see van Bergen et al., 2017; Hart et al., 2021; Torppa et al., 2022). Furthermore, HLE and parents’ concerns may also be related to parents’ reading history. Future research should consider these potential factors when examining the relationship between HLE and children’s literacy skills. Finally, the study did not include measures of children’s interest in literacy activities and reading independently, both of which have been demonstrated to be influential in early reading development (Martini and Sénéchal, 2012; Silinskas et al., 2020; Georgiou et al., 2021; Li and Li, 2022).

## 5. Conclusion

The present study examined the reciprocal associations between HLE and children’s reading skills in Hiragana and Kanji in a sample of Japanese parent–child dyads from kindergarten to Grade 3. The results show that Japanese parents may be sensitive to both their children’s reading performance and social expectations for school achievement and adjust their involvement accordingly during the transition period from kindergarten to early primary grades. The results further suggest that ALR may be associated with early reading development in both Hiragana and Kanji. These findings provide further evidence for the roles of HLE, parental awareness, and responsiveness to children’s performance. Future studies should consider the education system and social expectations for children’s achievement in examining the relationship between HLE and early reading acquisition.

## Data availability statement

The raw data supporting the conclusions of this article will be made available by the authors, without undue reservation.

## Ethics statement

The studies involving human participants were reviewed and approved by Ethics Committee, Graduate School of Education, Okayama University. Written informed consent to participate in this study was provided by the participants’ legal guardian/next of kin.

## Author contributions

TT and TI substantially contributed to the study conceptualization, data analysis, and interpretation. TT substantially contributed to the manuscript drafting. All authors critically reviewed and revised the manuscript draft and approved the final version for submission.

## Funding

This research was funded by grants from the Japan Society for the Promotion of Science (JSPS18K1322000 and JSPS22H01033) to TT.

## Conflict of interest

The authors declare that the research was conducted in the absence of any commercial or financial relationships that could be construed as a potential conflict of interest.

## Publisher’s note

All claims expressed in this article are solely those of the authors and do not necessarily represent those of their affiliated organizations, or those of the publisher, the editors and the reviewers. Any product that may be evaluated in this article, or claim that may be made by its manufacturer, is not guaranteed or endorsed by the publisher.
